# From advisory to action: the case for preserving PACCARB in the fight against antimicrobial resistance

**DOI:** 10.1017/ash.2026.10870

**Published:** 2026-09-25

**Authors:** Leila S. Hojat, Muhammad Dhanani, Amy Y. Kang, Salome O. Chitavi, Robert A. Weinstein, Edward Stenehjem

**Affiliations:** 1 Emory University School of Medicine, USA; 2 Northwestern University, USA; 3 Chapman University School of Pharmacy, USA; 4 Independant, USA; 5 Rush University, USA; 6 University of Colorado School of Medicine: University of Colorado Anschutz Medical Center, USA

## Abstract

The Presidential Advisory Council on Combating Antibiotic-Resistant Bacteria (PACCARB) was established to advise the government on strategies to mitigate antimicrobial resistance (AMR) and its increasing threat to public health and national security. Its members included experts from diverse disciplines such as healthcare, veterinary medicine, industry, public health, and agriculture who were positioned to interface with key stakeholders and the public in an aspirational multidisciplinary approach to combating AMR. The council was successful in elevating AMR as a national priority and played a crucial role in watershed events for antimicrobial stewardship (AS) such as the development of the second National Action Plan for Combating Antibiotic-Resistant Bacteria and the requirement for hospitals to establish antimicrobial stewardship programs (ASPs). Additionally, PACCARB was responsible for reframing the discourse on AMR through the context of One Health and evolved domestic efforts into global collaboration. While PACCARB’s future remains uncertain, stakeholder organizations must continue to advocate for policy in favor of AMR surveillance and prevention to avoid losing the momentum which PACCARB generated over the course of the last decade.

## Introduction

Antimicrobial resistance (AMR) is an urgent global public health crisis, directly causing an estimated 1.27 million deaths and contributing to nearly 5 million deaths worldwide.^1^ The Centers for Disease Control and Prevention (CDC)’s 2019 Antibiotic Resistance Threats Report estimates that antimicrobial-resistant pathogens in the United States (US) are responsible for more than 2.8 million infections and over 35,000 deaths each year.^2^ Given this substantial burden, sustained and coordinated action is essential across human, animal, and environmental health. In the US, this recognition led to the establishment of a dedicated federal advisory body to help guide a unified national response to AMR.

In 2014, a Presidential Executive Order established the Presidential Advisory Council on Combating Antibiotic-Resistant Bacteria (PACCARB) to advise the US government on policies and programs related to AMR, AS, surveillance of drug-resistant pathogens, novel diagnostics, infection prevention, and innovation.^3^ It has since issued detailed recommendations to human, animal, and environmental organizations and provided a public forum for dialog among federal agencies, clinicians, industry, the community, and other stakeholders. For antimicrobial stewards and epidemiologists, PACCARB’s work helped elevate AMR as a national priority and supported the policy environment underpinning AS and infection prevention activities.

As of August 2026, PACCARB has formally reconvened once; however, the viability of the council in the current administration remains in question. This development comes at a time of broader restructuring of public health and advisory structures and growing societal polarization around infectious diseases.

In this commentary, we (1) briefly review PACCARB’s history and unique role in the AMR response; (2) summarize stakeholder perspectives, including from current and prior PACCARB members; and (3) outline a call to action for the infectious diseases and stewardship communities to help ensure that national and global momentum against AMR is maintained and reevaluated independent of PACCARB.

## Establishment of the council

### Context

**Figure 1.**
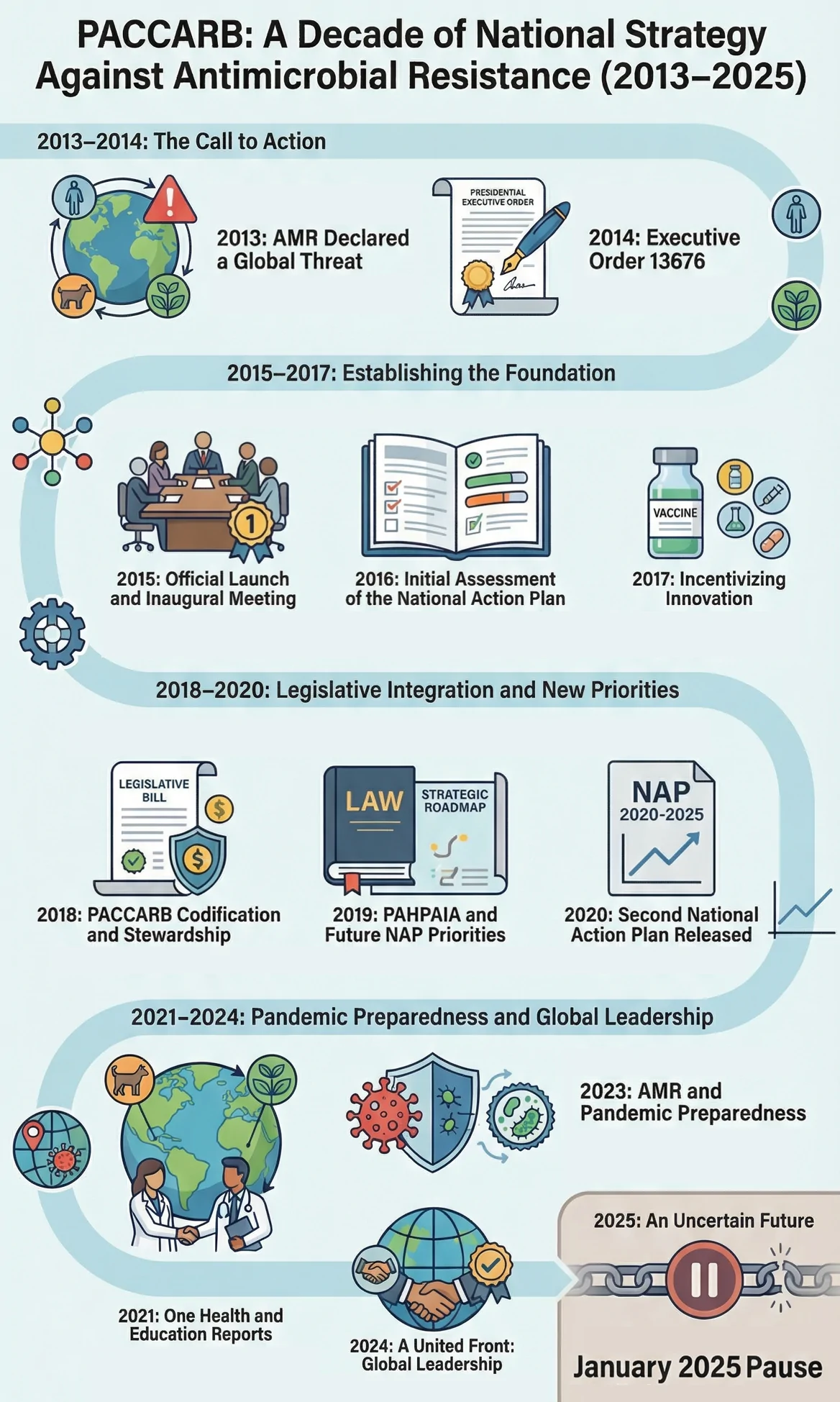
Figure 1 long description. Timeline of key events related to the formation of PACCARB and the major contributions and areas of influence leading up to its pause in January 2025.

The emergence of PACCARB was rooted in a period of escalating global and domestic awareness regarding the threat of AMR, with landmark declarations issued from the CDC and the World Health Organization (WHO) identifying AMR as one of the most serious threats to modern health systems. The CDC’s initial Antibiotic Resistance Threats report in 2013 provided the first comprehensive snapshot of the burden of resistant pathogens in the US, catalyzing federal and industry leaders to seek a coordinated response (Figure 1).^4^ This momentum led the President’s Council of Advisors on Science and Technology (PCAST) to release a 2014 report offering practical recommendations to strengthen the nation’s ability to combat the rise in antibiotic-resistant bacteria, which served as the blueprint for subsequent executive action.

### Initial formation and mandate

In September 2014, Executive Order 13676 was issued to direct federal agencies to implement the PCAST recommendations. This order established a dual structure for national leadership: the federal agencies of the Interagency Task Force for Combating Antibiotic-Resistant Bacteria and PACCARB.

PACCARB was designed as a body of external experts tasked with providing advice, information, and recommendations to the US Secretary of Health and Human Services (HHS). The council’s authority was further solidified via inclusion in the Pandemic and All-Hazards Preparedness and Advancing Innovation Act of 2019 (PAHPAIA), which provided statutory authority for the council to advise the HHS Secretary regarding programs and policies to reduce AMR.^5^

PACCARB held its inaugural public meeting in September 2015, marking the start of its role as a principal “architect” of the AMR movement. From its inception, PACCARB occupied a unique position in the federal landscape, originating at the highest levels of government, which granted it credibility and national visibility. Crucially, it was established as the designated government entity dedicated to addressing AMR through a One Health lens, acknowledging that the health of humans, animals, and the environment are inextricably linked.

## Structure and scope

### A unique platform for stakeholder engagement

PACCARB’s operational model was defined by transparency and direct stakeholder engagement. The council’s membership structure was intentionally diverse to support this mission, with approximately 30 voting and non-voting members collectively representing the full One Health spectrum. By holding multiple public meetings each year, the council provided a rare platform where subject matter experts from academia, health systems, industry, agriculture, public health, and veterinary medicine could interface directly with federal officials. Beyond standing members of the council and its leadership, PACCARB included rotating organizational liaisons with broad representation (Table 2). These stakeholders provided key input to PACCARB discussions, reflecting the interest organizations outside hospital-based human health held in the actions taken by PACCARB. Additionally, meetings were attended by guests representing a broad range of fields such as biotechnology and patient safety. PACCARB also prioritized the “voice of the patient,” actively seeking stories from individuals directly impacted by resistant infections to inform policy recommendations. These meetings fostered open dialog and allowed the council to catalyze innovations, such as through “Innovation Spotlight” sessions for industry startups.

**Table 1.** Exemplar quotes pertaining to major themes identified in the survey Table 1 long description.

Theme	Exemplar quotes
**Theme 1: Elevated importance of AMR**	AMR [shifted] from a single and limited focus to the perspective that AMR is a silent, expanding epidemic that could result in the next global pandemic. It [needed] to be recognized as a threat to national security, potential economic disaster with significant human and societal burdens.
	Thinking of AMR as a national security issue helped to align AMR actions with other government priorities and gained efficiencies.
	The US effort on AMR was placed in a global context because of the PACCARB’s work and sharing it with the world.
	Encouraging/driving actions for the initial US National Action Plan and the creating its 2018 update were at the heart of the project. I also thought the work on holding public discussions about incentives for antibiotics and diagnostics was very high impact.
**Theme 2: Embracing one health**	[There was a] shift from strictly a human health focus to the inclusion of multiple groups, organizations, professions, and disciplines under a One Health approach.
	We became the exemplar in uniting One Health domains and establishing a sense of comradery among all opinions.
	[PACCARB moved] the needle towards a One Health approach to the issue [of AMR].
**Theme 3: Multidisciplinary approach**	[PACCARB] offered the public, the different agencies, and actors in the government the chance to get together and discuss things. From CMS to DoD and so on. That was the major strength.
	[PACCARB’s] strength was from its broad base of expertise and disciplines that mirrored critical players involved and impacted by AMR; it became a scientific cross-pollination effort to create recommendations beyond any single discipline.
	I had never been in the room with a veterinarian. I had never talked about the pork industry. It was great to speak to experts in these fields and have face-to-face discussions. You understand people better if you are there in the same room speaking to them in person.
**Theme 4: Advisory role as a limitation**	I couldn’t go to HHS and make them go do something because it would be good for […]. That wasn’t possible.
	[PACCARB] lacked ability and authority to implement actions and its clout came from an advisory role.
	Products required buy-in from US [government] agencies and strong promotion… sometimes lip-service only [was given], needed real action.

**Table 2.** Examples of organizational liaisons and guests attending PACCARB meetings and the sectors represented Table 2 long description.

Attendee	Sector
**Organizational liaisons**	
Animal husbandry	Pork Producers Council, National Turkey Federation, North American Meat Institute
Professional organizations representing bedside providers	American Nurses Association, Society of Infectious Diseases Pharmacists, Pediatric Infectious Diseases Society
Professional organizations within non-hospital-based fields of human health	Association of State and Territorial Health Officials, Association for Public Health Laboratories, National Association of Directors of Nursing Administration in Long Term Care
Animal health	American Association of Extension Veterinarians, Animal Health Institute
Global non-profits	Bill & Melinda Gates Foundation, Wellcome Trust, Pew Charitable Trusts
Industry	EpiX Analytics, Direct Meds Incorporated
**Guests**	
Animal health	Food Animal Concerns Trust, Keep Antibiotics Working
Biotechnology firms	Becton Dickinson, T2 Biosystems, Phagelux
Non-profits	Doctors Without Borders, National Resources Defense Council
Professional organizations of health professionals	Infectious Diseases Society of America, Society for Healthcare Epidemiology of America, American Thoracic Society
Patient safety organizations	Nile’s Project, Mothers Against Medical Error, Consumer Reports, Patient Safety Project
**Non-voting ex officio members**	
Federal agencies	Department of Health and Human Services, Centers for Disease Control and Prevention, Food and Drug Administration, National Institutes of Health, Department of Agriculture, Department of Defense

PACCARB also included non-voting ex officio members from federal agencies to contribute governmental expertise and ensure coordination with national programs like the Interagency Task Force. Finally, sociologists and economists were essential participants given the extent to which human behavior was recognized as affecting AMR, as were representatives of private enterprises who could discuss the development of innovative tools to address AMR. Together, this blend of independent experts and federal representatives ensured that PACCARB’s recommendations reflected a wide range of viewpoints and practical knowledge spanning human health, animal health, and environmental considerations.

### Scope of work

PACCARB formally convened on 26 occasions between its September 2015 inaugural meeting and August 2024. Over this period of nearly a decade, PACCARB’s work spanned a broad range of critical and emerging topics. Central to its mission was the oversight and development of the NAP, described by the Food and Agriculture Organization of the United Nations (FAO) as the “national cross-sectoral action plan of the United States of America.” The US had committed to developing a NAP in accordance with the Global Action Plan (GAP) on AMR adopted by United Nations (UN) member states at the 2015 World Health Assembly, and these models were later established to be effective in promoting more judicious antimicrobial use.^6^ While the Interagency Task Force was responsible for implementing the NAP, the council provided the initial assessments of the 2015 plan and later identified the priority areas that shaped the subsequent 2020–2025 iteration.

The council’s output was characterized by detailed recommendation reports and resolution letters that addressed both long-term strategies and urgent needs. Key areas of influence included:

- Interprofessional Education: Strategies for advancing education and practice to combat AMR across healthcare disciplines and venues.
- Antimicrobial Access and Use: Recommendations for improving access and AS across the One Health spectrum.
- Economic Incentives: Proposals for incentivizing the development of new vaccines, diagnostics, and therapeutics to fix the “broken” antibiotic pipeline.
- Pandemic Preparedness: A significant focus in later years, exploring the intersection of AMR and viral pandemics, such as COVID-19, to ensure future preparedness plans integrate AMR threats.

## Stakeholder perspectives

To complement the scope and output of PACCARB as defined in official records, the authors surveyed key stakeholders to elicit a first-hand narrative of the council’s role, strengths, and perceived impact.

### Survey development and dissemination

Individuals were identified as current or former PACCARB members via public record of the PACCARB roster and public meeting minutes.^7^ A total of 37 current and former PACCARB members were identified, 17 of which were contacted based on availability of contact information and referrals from initial participants. Individuals were involved in PACCARB during different stages of its development and represented diverse fields including adult and pediatric infectious diseases, veterinary medicine, policy development, and public health. Additional affiliations included academic institutions, medical centers, the pharmaceutical industry, and global One Health Community.

The authors developed a survey to gather perspectives on PACCARB-related topics from primary stakeholders. The survey questions were designed to elicit background information on the stakeholder’s training and involvement in PACCARB and to gather thoughts regarding the advisory council’s role, strengths, weaknesses, successes, and overall impact. The final question explicitly asked why PACCARB should continue. The survey was disseminated via email, and multiple formats for response were offered: writing responses in a document and returning via email; completing the survey online via a link; or discussing the questions with one of the authors in a structured interview.

## Results

Of the 17 individuals contacted, 7 completed the survey and/or an interview. Several others responded that they preferred not to provide comments due to the current unresolved status of the committee. Respondents represented the full spectrum of PACCARB’s diverse membership. Comments were aggregated and used to inform the following sections on PACCARB’s history, impact, and importance. Given the low survey response rate and convenience sampling method of participant identification, results were considered illustrative rather than comprehensive in terms of assessment of committee member perspective.

Responses revealed similar themes across the participants in relation to their experience and impression of PACCARB (Table 1). These themes included how PACCARB elevated the importance of AMR at the national, state and organizational levels; embraced and advocated for a One Health approach to curb AMR; emphasized the importance of multidisciplinary stakeholder involvement; and described the inherent limitations of an advisory board.

### Elevated importance of AMR (Theme 1)

A central theme across survey responses was the role PACCARB played in elevating the importance of AMR, reframing it as an urgent, large-scale threat with significant human and societal burdens. Assessment of the NAP was referenced by most respondents and considered fundamental to promoting AMR as an issue of national importance. Because of PACCARB, AMR moved from a narrow problem confined to a single area of medicine to a broader issue associated with public health, national security, and economic ramifications. Discussion of human case studies also provided a way of humanizing AMR to the public, emphasizing the importance of education and science literacy. In more recent years, the council made strides in articulating the economic case for AMR action, evolving its valuation frameworks to better reach policymakers.

### Embracing One Health (Theme 2)

A second theme identified in the survey was PACCARB’s strategic transition and consensus building toward a One Health framework, integrating human, animal, agriculture, and environmental perspectives, and shifting from a biomedical focus to a more holistic and multi-sectoral dynamic approach to understand and address the threat of AMR. This included advocacy efforts for increasing stewardship in both clinical and agricultural settings. PACCARB’s engagement of experts across disciplines was considered a key structural strength which facilitated embracement of One Health, though bridging the gap between human and animal health concerns (e.g., related to commercial animal husbandry) remained an ongoing challenge due to competing priorities and limited oversight of the agricultural sector.

### Multidisciplinary stakeholder involvement (Theme 3)

The primary strength of PACCARB lay in its inclusive and multidisciplinary composition. Its voting members included experts drawn from adult and pediatric infectious disease medicine, public health, microbiology, veterinary medicine, environmental health, agriculture, diagnostics, and the pharmaceutical and biotechnology industries, strategically fostering collaboration across traditionally siloed fields to enable a shared understanding of each group’s incentives and disincentives. This diversity also allowed the council to build strong connections between organizations and recognize the importance of business partnerships in sustaining the antibiotic pipeline. While respondents acknowledged that reaching consensus across sectors and past disparate groups could be challenging, PACCARB members fostered an environment characterized by mutual respect and shared purpose, leading to more effective consensus-building, collective action, and productivity.

### Advisory role as a limitation (Theme 4)

While many strengths were identified, many respondents viewed the advisory-only role of PACCARB as a major constraint on implementation. PACCARB’s advisory role meant that recommendations required buy-in from federal agencies and other stakeholders, which respondents described as variable and thus limited the council’s direct impact on policy execution. A more proactive relationship with Congress was sought after, particularly related to the concepts of investing in the antibiotic pipeline and considering antibiotics as a public good, but the opportunity to influence legislation was generally limited. The inability to directly implement or enforce recommendations was seen as a severe structural limitation, and a unique challenge for an advisory body operating within complex policy environments.

## Major contributions and importance

Survey respondents and review of online PACCARB materials identified several areas to which PACCARB had made meaningful contributions, including sustaining visibility and attention on AMR and maintaining AMR as a policy priority. PACCARB’s formal outputs, such as meeting notes, presentations, reports, and recommendations, were perceived as valuable contributions that informed national strategy, influenced legislation, and reinforced interprofessional education among federal, state, human and animal health organizations. PACCARB also pushed the boundaries of the AMR conversation by introducing new subject arenas, including advocacy for addressing antifungal resistance, the role of the microbiome, the impact of the environment and agriculture on the spread of resistance, and development of regulatory frameworks.

Furthermore, the council influenced regulatory frameworks. In response to Task Letters from HHS, PACCARB formed working groups and subcommittees to develop materials which ultimately informed 13 formal Recommendation Reports.^8^ Several of PACCARB’s recommendations can be linked to downstream policies and actions which served as critically important drivers to combat AMR.

**Figure 2.**
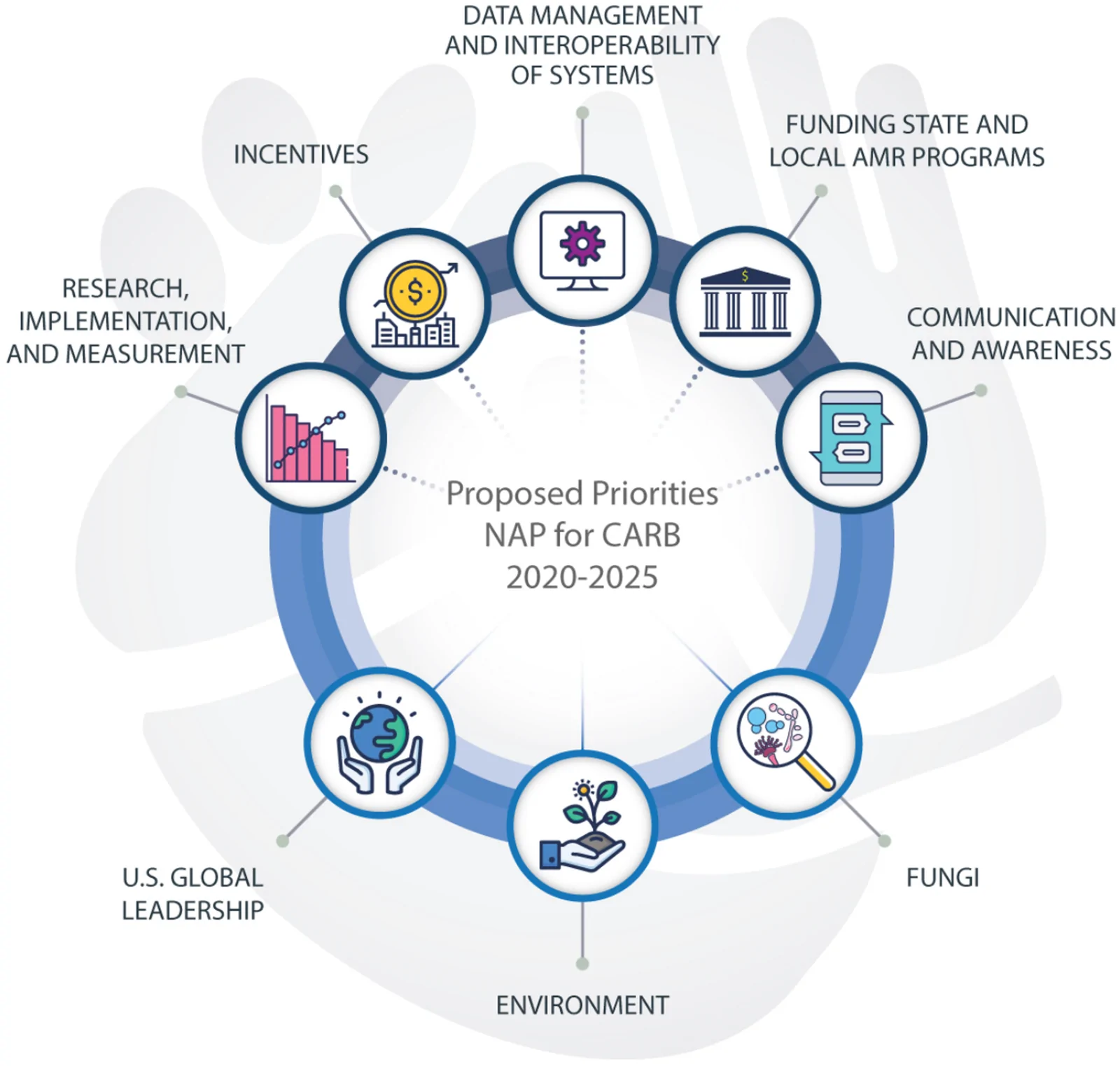
Figure 2 long description. Diagram included in the PACCARB recommendation report 7 executive summary demonstrating the proposed five priorities (top) within three content areas (bottom) recommended to be included in the 2020–2025 National Action Plan. Public domain.

- Recommendation Report 6 and resolution letter to the HHS Secretary led to the finalization of the 2019 CMS Conditions of Participation requiring ASPs to be established across all hospitals, notably including critical access facilities and nursing facilities.
- Recommendation Report 7 informed the 2020–2025 iteration of the NAP, outlining 17 priorities across 5 goals, fulfilling the goal described in the 2015 GAP on AMR (Figure 2).^9^
- Recommendation Report 10 supported the passage of the Developing an Innovative Strategy for Antimicrobial Resistant Microorganisms (DISARM) Act and the Pioneering Antimicrobial Subscriptions to End Upsurging Resistance (PASTEUR) Act to incentivize the development and appropriate use of novel antimicrobial agents.
- Recommendation Report 12 identified 9 US recommendations to support 4 global priorities for AMR, later reflected in a declaration from the 2024 UN High-Level Meeting on AMR.

PACCARB’s influence extended beyond US borders, positioning the US as a global leader in the fight against AMR. The council participated in high-level meetings at the United Nations General Assembly and collaborated with international bodies like the WHO, World Organization for Animal Health (WOAH), and FAO. Its most recent major report, A United Front: Collaborative Global Leadership to Combat Antimicrobial Resistance, emphasized this international commitment. Its multidisciplinary model was frequently cited as an exemplary framework for other nations; notably, it served as a reference for the establishment of the WHO’s Independent Panel for Evidence for Action against AMR (IPEA).

## Conclusions and call to action

PACCARB has functioned as a critical guiding coalition that elevated AMR to a national priority, fostered unprecedented transparency in federal policy, and provided a comprehensive One Health framework for the US response. For the infectious diseases and antimicrobial stewardship communities, PACCARB has served as a vital forum where gaps in the national response were made apparent and joint actions between government and industry were facilitated. Its current uncertain status threatens the dismantling of decades of momentum built against an ever-evolving public health crisis. Further, other emerging societal threats could worsen AMR such as vaccine hesitancy, disinformation, the dismantling of public health entities such as the CDC’s Healthcare Infection Control Practices Advisory Committee (HICPAC), the loss of healthcare coverage among millions of Americans, and diminishing trust in science.

As PACCARB’s future is defined, infectious diseases stakeholder organizations such as IDSA, PIDS, and SHEA will need to increase their advocacy efforts to ensure the accumulated infrastructure and expertise is not lost. Such advocacy efforts are those in favor of AMR surveillance and prevention, including increasing support for infection prevention and AS, as well as legislation. The PASTEUR Act is a representative example, which was recently reintroduced by a bipartisan group to the US House of Representatives and seeks to establish a subscription model to encourage development of novel antimicrobials.

Another element which may protect PACCARB is identification of shared priorities with respect to the current administration. Admittedly, the individual choice narrative contrasts sharply and dishearteningly with much of PACCARB’s mission and the concept of antimicrobials as a public good. However, integrating language from government healthcare initiatives into PACCARB’s discourse, such as confronting chronic disease, targeting preventive care, focus on the microbiome, limiting antimicrobial overuse in agriculture and pediatrics, and increasing transparency could provide a conduit through which the committee’s main agenda could reach policymakers.

In considering these options, it is worth contextualizing PACCARB’s achievements against persistent realities. Despite developing expansive national strategies, action plans, white papers, guidelines, societal recommendations, and international declarations, rates of AMR continue to increase. While these efforts likely prevented an even worse trajectory of AMR progression, it is prudent to reflect on what approaches have and have not worked. One possibility is that the comprehensiveness of prior action plans has itself been a limiting factor, with extensive priorities diluting resources and preventing meaningful change. The 2020–2025 NAP has reached its end date, and a critical near-term role for PACCARB could be a structured evaluation of progress on that plan and the development of targeted, actionable recommendations to inform the next iteration, prioritizing depth over comprehensiveness.

Advances in technology and therapeutics will also shape the next chapter of AMR in which PACCARB could continue to have a meaningful role in. Clinical decision support tools that guide prescribing at the point of care represent an immediately actionable lever. Longer-term, emerging strategies such as phage therapy, microbiome manipulation and novel delivery antimicrobials delivery platforms may expand the therapeutic armamentarium, though widespread clinical application remains limited.

Lastly, establishing additional entities with concurrent messaging may serve both to amplify the AMR agenda as well as introduce a protective level of redundancy. The AMR Strategic Coalition (ASC) was established in September 2025 to unite a multidisciplinary and multisectoral group of experts to generate policy solutions to advance AMR efforts and emphasize principles of One Health. Their first major contribution was a perspective piece delivered to the WHO in anticipation of the GAP on AMR set to be developed in 2026, and their aim is to link US expertise to global partners in this space. Independent organizations such as ASC increase the resilience of the fight against AMR, ensuring that the continued public awareness of its urgency is not disrupted, regardless of political circumstances.

## Figure 1. Long description

The timeline spans from 2013 to 2025, detailing the formation and activities of PACCARB. Key events include: 2013: WHO and CDC declare AMR a global threat. 2014: Executive Order 13676 establishes PACCARB. 2015: Official launch and inaugural meeting. 2016: Initial assessment of the National Action Plan. 2017: Incentivizing innovation. 2018: PACCARB codification in legislation. 2019: PAHPAIA and future NAP priorities. 2020: Second National Action Plan released. 2021: One Health and introduction of PASTEUR Act. 2023: Pandemic preparedness in the era of AMR. 2024: A united front with global leadership. 2025: An uncertain future.

Navigate back to Figure 1..

## Table 1. Long description

A table with four themes and exemplar quotes from a survey on PACCARB’s impact on AMR. The table has two columns: Theme and Exemplar quotes. Theme 1: Elevated importance of AMR. Row 1: AMR shifted from a silent epidemic to a national security threat. Row 2: AMR as a national security issue aligned actions with government priorities. Row 3: US effort on AMR was recognized globally. Row 4: Initial US National Action Plan and updates were central to the project. Theme 2: Embracing one health. Row 1: Shift to a One Health approach including multiple groups. Row 2: PACCARB united One Health domains. Row 3: PACCARB moved towards a One Health approach. Theme 3: Multidisciplinary approach. Row 1: PACCARB offered a platform for various agencies to discuss AMR. Row 2: PACCARB’s strength was its broad base of expertise. Row 3: Face-to-face discussions improved understanding. Theme 4: Advisory role as a limitation. Row 1: PACCARB could not mandate actions. Row 2: PACCARB lacked authority to implement actions. Row 3: Products required buy-in from US agencies.

Navigate back to Table 1..

## Table 2. Long description

A table with 10 rows and 2 columns. The first column lists categories of organizational liaisons and guests, and the second column lists specific organizations or groups within each category. Categories include Animal husbandry, Professional organizations representing bedside providers, Professional organizations within non-hospital-based fields of human health, Animal health, Global non-profits, Industry, Guests, Biotechnology firms, Non-profits, Professional organizations of health professionals, Patient safety organizations, and Non-voting ex officio members. Each category lists multiple specific organizations or groups.

Navigate back to Table 2..

## Figure 2. Long description

A circular diagram representing proposed priorities for the National Action Plan for Combating Antibiotic-Resistant Bacteria (CARB) from 2020 to 2025. The diagram is divided into three main content areas: Research, Implementation, and Measurement; Incentives; and U.S. Global Leadership. Each content area is further divided into specific priorities. The top section includes Data Management and Interoperability of Systems, Incentives, Research, Implementation, and Measurement, Funding State and Local AMR Programs, and Communication and Awareness. The bottom section includes U.S. Global Leadership, Environment, and Fungi. Each priority is represented by an icon and is connected in a circular flow, indicating the interconnected nature of these priorities.

Navigate back to Figure 2..
